# Fibrolipome du pouce

**DOI:** 10.11604/pamj.2015.22.120.7901

**Published:** 2015-10-12

**Authors:** Badr Ennaciri, Mohamed Ouadghiri

**Affiliations:** 1 Service de Chirurgie Orthopédique, CHU Avicenne, Rabat, Maroc

**Keywords:** Fibrolipome, tumeur de la main, microchirurgie, Fibrolipoma, tumor of the hand, microsurgery

## Image en medicine

Les tumeurs nerveuses représentent 5% des tumeurs de la main, le schwanome et le neurofibrome sont les plus fréquents. Le fibrolipome est une tumeur bénigne très rare, d'origine inconnue, et qui se développe à partir des nerfs périphériques. Macroscopiquement, il se présente sous forme d'une formation lobulée avec composante graisseuse et tissulaire. Microscopiquement, cette tumeur est caractérisée par la présence de tissus adipeux et conjonctif infiltrant les enveloppes du nerf. L'exérèse chirurgicale fait appel aux procédés de microchirurgie. Nous rapportons l'observation d'une patiente âgée de 62 ans, sans antécédents pathologiques notables, admise pour tuméfaction à la racine du pouce évoluant depuis 2 ans. L'examen clinique à l'admission avait objectivé une masse de 2cm de diamètre, arrondie, circonférentielle, intéressant la racine du pouce gauche, mobile par rapport aux plans profond et superficiel et sans signes inflammatoires en regard. Le bilan radiologique n'avait pas montré d'atteinte osseuse. L’échographie du pouce gauche avait objectivé une formation sous cutanée des versants palmaire et dorsal avec contours lobulés hypo-échogène finement hétérogène, avasculaire au Doppler couleur, mesurant 8 mm d’épaisseur. Par abord palmaire à la racine du pouce, nous avons mis en évidence une formation polylobée avec composante graisseuse et tissulaire adhérente au nerf collatéral radial du pouce; après dissection du pédicule vasculo-nerveux, l'exérèse du fibrolipome était facile. L’évolution était satisfaisante sans récidive locale ni troubles sensitivomoteurs.

**Figure 1:**
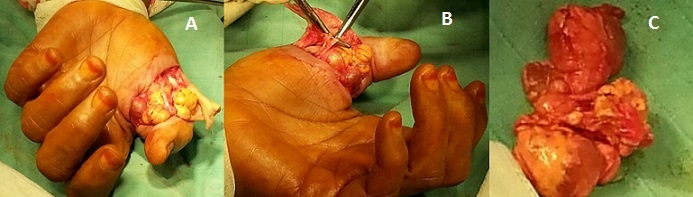
(A) formation polylobée graisseuse et tissulaire à la racine du pouce; (B) dissection du pédicule vasculo-nerveux adhérent au fibrolipome; (C) volumineuse tumeur après résection complète avec double composante adipeuse et tissulaire

